# ALA-based photodynamic priming in murine skin increases blood flow and oxygenation

**DOI:** 10.1117/1.JBO.30.S3.S34106

**Published:** 2025-09-13

**Authors:** Aleksandra Ilina, Marien Iliza Ochoa Mendoza, Xu Cao, Tayyaba Hasan, Brian W. Pogue

**Affiliations:** a University of Wisconsin School of Medicine and Public Health, Department of Medical Physics, Madison, Wisconsin, United States; b Massachusetts General Hospital, Harvard Medical School, Wellman Center for Photomedicine, Boston, Massachusetts, United States

**Keywords:** photodynamic therapy, tissue oxygenation, 5-aminolevulinic acid, protoporphyrin IX, blood flow

## Abstract

**Significance:**

Topical photodynamic therapy (PDT) with protoporphyrin IX (PpIX) converted from 5-aminolevulinic acid (ALA) is a well-established noninvasive method of treating skin conditions and lesions. During PDT, there can be response dynamics within the tissue that are affected by the light delivery, seen with fractionated delivery and in subcurative priming delivery. Fractionated light doses can considerably increase efficacy of 5-ALA PDT response.

**Aim:**

We aim to examine the changes in physiological blood flow, tissue oxygenation, and PpIX concentration during and after light delivery in topical ALA-PDT in nude mouse skin.

**Approach:**

We compared three schemes of light delivery for topical ALA-PDT in nude mice, including (1) full light delivery without fractionation, (2) two equal fractions (50% and 50%) of light separated by 2 h, and (3) a 5% light dose fractionation by 2 h prior to the main 95% light dose. Tissue oxygen imaging was assessed with the hypoxia signal from delayed fluorescence of PpIX itself within the tissue, as well as by confirmation with Oxyphor phosphorescence lifetime quenching imaging.

**Results:**

The results of blood flow imaging and hypoxia imaging from PpIX and oxygen imaging with Oxyphor each showed evidence of increased capillary flow and tissue oxygenation after the initial 5% light dose, increased at the side of irradiation. This increased capillary flow and tissue oxygenation are presumably from vasodilation and local capillary flow increase. PpIX replenishment occurs during the intervening dark period after the initial light delivery.

**Conclusion:**

These observations suggest that increasing oxygen and capillary flow combined with increased PpIX production together yield increased PDT efficiency, amplified by this initial light dose from a photodynamic optical priming event occurring 2 h prior to full PDT light delivery.

## 1 Introduction

Photodynamic therapy (PDT) is well known to be a treatment that kills tissue with a mixture of acute biochemical and biophysical mechanisms, and the complex tissue milieux can be altered even during the active light delivery. The changes can be so dynamic in time that there are blood flow alterations during the treatment^1^^–^^3^ or photosensitizer changes from redistribution^4^ or bleaching,^5^^–^^7^ each of which can alter the pattern of damage, either amplifying or suppressing it depending upon the photosensitizer type,^8^ optical dose rate, and tissue type. An early exploration of this alteration was especially discovered in fractionated delivery,^9^^–^^12^ where separation of delivery into light fractions delayed by hours was shown to amplify the treatment. More recently, studies have demonstrated how low level PDT can alter the biochemical signaling and “prime” the tissue, such as a neoadjuvant treatment, to amplified damage from a secondary treatment. In this study, blood flow and oxygenation changes present in tissue from PDT with aminolevulinic acid (ALA) were examined to help explain some of the biological changes seen from low level and fractionated delivery.

Fractionation of ALA-based PDT in mouse skin was extensively studied and developed by de Bruijn et al.,^13^^–^^16^ showing that a small priming of 5% to 10% of the light dose given 75 min prior to the remainder of the light dose could significantly amplify the observed damage, compared with delivery of all the light in a single dose. This observation was unique to ALA and not methyl-ALA,^17^ and the mouse skin was used as a model in these studies due to its high production of protoporphyrin IX (PpIX). These basic observations were translated to human cancers^18^ and ultimately showed a dramatic improvement in lesion clearing in a 5-year randomized, prospective study.^19^ Although the efficacy is clear, the exact biophysical mechanisms have always been less clear and the relationship to photodynamic priming has not been explored systematically.

Photodynamic priming (PDP) has been a more recent offshoot of these studies on fractionation,^20^^–^^22^ where the more subtle effects of PDT have been shown to amplify the response of secondary therapies. PDP induces changes to the cellular, stromal, and vascular microenvironment of tissue, making it more receptive to subsequent additional therapies including chemo- and immunotherapy.^23^ The entire mixture of changes that occur is not well documented yet, but structural alterations in pancreatic cancer have been documented from CT scans,^24^ and blood flow alteration is well documented from several photosensitizers.^8^^,^^25^^–^^28^ However, what has not been fully explored to date is the combination of blood flow and oxygenation changes that occur in tissue, and how this is dynamic with the magnitude of the light delivered. This parameter space is further examined here using ALA-PpIX in murine skin. This work has been facilitated by a recent discovery that the delayed fluorescence from PpIX itself can be a direct reporter of the lack of oxygen in tissue, as the signal increases with the onset of hypoxia.^29^^–^^32^ So, in this work, measures of blood flow change were combined with those of hypoxia and photosensitizer concentration, all taken diagnostically *in vivo* without any alteration or contact with the tissue.

Perhaps most importantly, in this study, the choice has been to examine ALA-PpIX because it is such a unique photosensitizer, which is potent but also dynamic in time and typically intracellular in location, and likely is the most widely used of all agents in humans today. PpIX is produced intracellularly,^33^ but can undergo redistribution to the interstitial and plasma spaces over time,^34^^–^^36^ and each of these can induce a uniquely different photodynamic effect based upon the biophysical location of where the PDT damage is being deposited. The value of ALA as a priming agent is very high for human therapy, given its widespread use as both a skin treatment^37^ and fluorescence-guided surgery of glioma^38^ and bladder cancers.^39^ There is high potential to use ALA-based PDP as a neoadjuvant treatment to amplify secondary therapies, and a better understanding of the blood flow and tissue oxygenation changes that occur in this will help determine the optimal ways to control this. This study examines these changes occurring from very mild fractionation, with just 5% light dose early, to medium fractionation, with a 50% light dose early. These changes are compared with the delivery of the full PDT light dose, and the blood and oxygen dynamics are tracked over time.

## 2 Methods

### 2.1 Animals

**Fig. 1:**
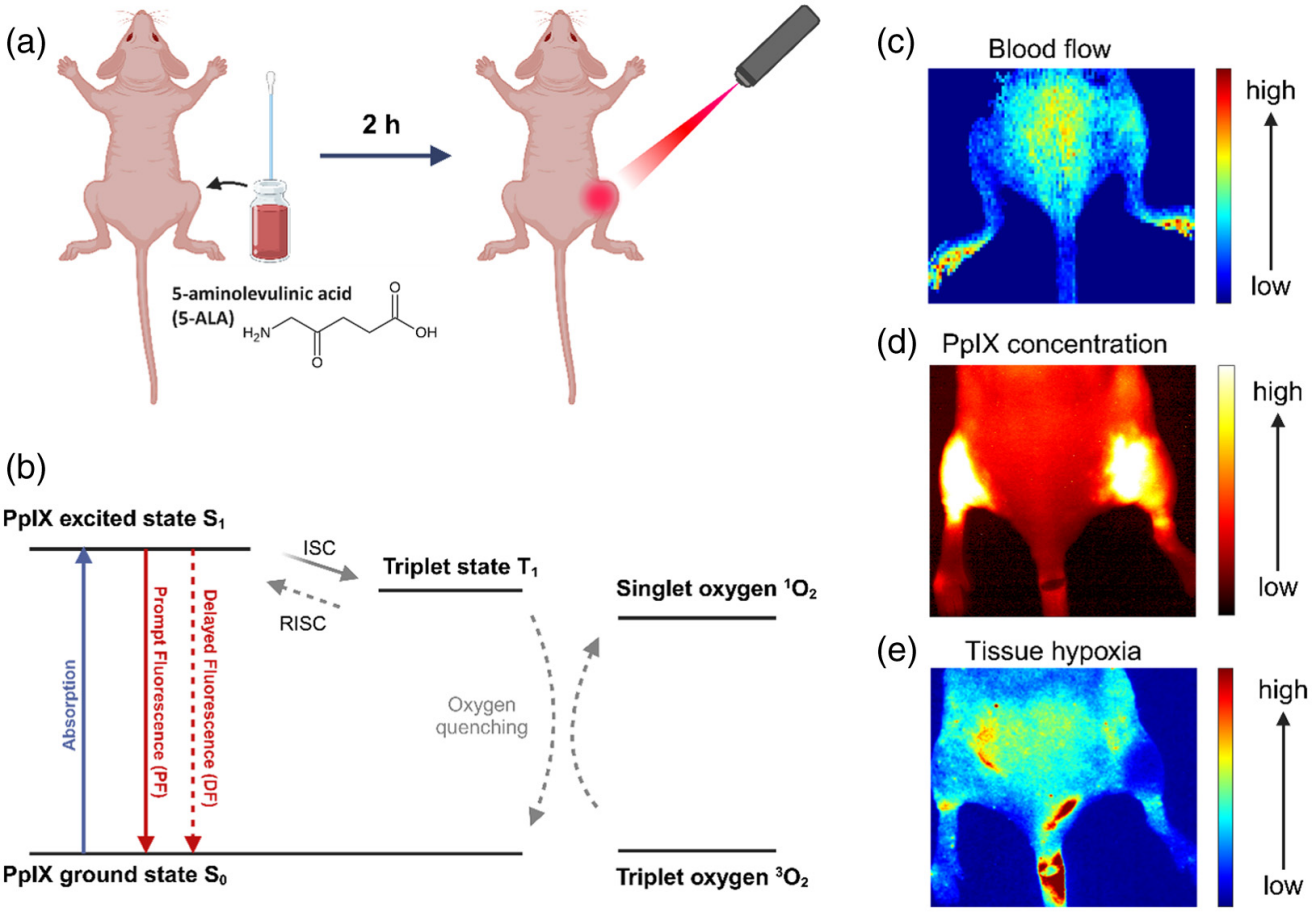
(a) Schematic representation of the PDT procedure. (b) Jablonski diagram showing the prompt and delayed fluorescence origin (ISC, intersystem crossing; RISC, reverse intersystem crossing). (c)–(e) Examples of the images for the blood flow (c), PpIX concentration (d), and tissue hypoxia (e).

The study completed was part of a vertebrate subjects protocol approved by the University of Wisconsin Animal Care and Use Committee (ACUC), and all procedures followed the approved protocol. A total of 15 athymic nude male mice (Envigo RMS, Allison Pointe, Indianapolis, IN) were used for the studies, supplied at 6 to 8 weeks of age and acclimatized for 1 week after delivery to our vivarium. Mice were placed on a low chlorophyll diet for 36 h prior to the treatment to reduce background autofluorescence. For the treatment, a solution of 20% ALA was applied to the top surface of both hind legs of each mouse. The mice were left under isoflurane anesthesia for 30 min after the ALA application to the skin to facilitate the absorption and the production of PpIX. After this period, the mice were returned to cages and kept separated in the dark for 2 h before the PDT treatment [Fig. 1(a)]. The mice were kept under 2.5% isoflurane anesthesia for the duration of the PDT treatment and the follow-up imaging and placed on a heating pad. After the treatment and imaging, the mice were returned to their separate cages and kept awake for the dark interval between the fractions if a fractionated approach was used. It should be noted that there are small differences in the animal model used compared with the use of SKH1 mice in previous studies.^9^^–^^12^

### 2.2 Light Treatment

For the PDT treatment, the total dose delivered for full PDT treatment was $100\;J/{\mathrm{cm}}^{2}$ at a fluence rate of $50\;\mathrm{mW}/{\mathrm{cm}}^{2}$, using a 635-nm laser (635-nm 100-mW pigtailed laser diode, Edmund Optics), delivered through a fiber to an achromatic collimator with adjustable focus (Thorlabs Inc., New Jersey). The spot size used was 1.0 cm in diameter, and the irradiation time was 2000 s for the full dose. Three irradiation schemes were used including

1. full light delivery without time fractionation ($100\;J/{\mathrm{cm}}^{2}$, for 100% PDT);
2. two equal fractions of $50\;J/{\mathrm{cm}}^{2}$ (50% and 50%) light separated by 2 h;
3. 5% light dose ($5\;J/{\mathrm{cm}}^{2}$) fractionation, 2 h prior to the remaining 95% ($95\;J/{\mathrm{cm}}^{2}$) light dose.

### 2.3 Diagnostic Measurements

The PpIX concentration was effectively measured by quantitative fluorescence imaging of the PpIX by a custom imaging setup. An emCCD camera (PI-MAX 4, Princeton Instruments) was employed to capture delayed fluorescence images using a 250-microsecond gate width after each pulse. Besides time gating, a 650-LP filter blocked the 635-nm excitation band, and the images represented PpIX’s delayed fluorescence, indicating tissue hypoxia.^40^ This tool is useful for direct online monitoring of ${\mathrm{pO}}_{2}$ changes during PDT. As we see from Fig. 1(b), the excited state of PpIX can undergo intersystem crossing to the intermediate excited triplet state. This state is usually quenched by the intracellular triplet oxygen, which is excited to produce singlet oxygen, which is the main agent in PDT. However, sometimes the intermediate states can undergo a reverse intersystem crossing, emitting a photon. Because of this process, there is a time delay between the light absorption and the following emission from the ground state is therefore much longer, causing the fluorescence to be “delayed.” Because the oxygen acts similar to a quencher to an intermediate triplet state, the delayed fluorescence (DF) lifetime is very sensitive to oxygen, and lower ${\mathrm{pO}}_{2}$ would mean longer DF lifetimes. Image acquisition occurred at 411 ms per frame, for a total of 500 frames. In addition, a dichroic mirror and iCCDs 1 and 2 (FLIR, Princeton Instruments) were used to capture white-light and prompt fluorescence images simultaneously with the delayed fluorescence. The same filter settings were applied for both prompt and delayed fluorescence. Prompt fluorescence, acquired during the excitation pulse, corresponds to PpIX location, with intensity related to its concentration. Each mouse dataset thus includes white-light, prompt, and delayed fluorescence images over 3.5 min. Measurements of the blood flow in the superficial layer of the skin were quantified using the tissue perfusion imager (${\mathrm{MoorO}}_{2}\mathrm{Flo}\text{-}2$, Moor Instruments, Delaware). The tissue perfusion imager and the PpIX imager were aligned to match the same field of view. The blood flow images were measured over 1.5 min for each leg of the mouse. The resulting images of DF, PF, and blood flow were averaged over time, the mean intensity value was calculated from the same region of interest on each figure, and control measures were taken from unirradiated skin areas contralateral to the treatment. Each set of images was taken prior to the treatment (2 h post-ALA application), immediately after first dose in case or the factionated scheme, or after a full treatment in case in single-dose treatment, after 2-h dark period for fractionated treatment, after the delivery of the second fraction of the dose, and 2 h post-treatment (after second dose for the fractionated treatment, or full dose for single-dose treatment)

Measurement of ${\mathrm{pO}}_{2}$ was accomplished via an oxygen-sensitive phosphorescence lifetime assay, using the Oxyphor PdG4 probe (Oxygen Enterprises, Philadelphia, PA), with the sensor positioned directly at the mouse leg. Emission lifetime was measured on an OxyLED phosphometer (Oxygen Enterprises, Philadelphia, PA) with a fiber optic measurement setup, and a sampling rate of ∼3  Hz, to quantify local changes in the ${\mathrm{pO}}_{2}$ levels during the PDT treatment.

Statistical analysis was performed for differences between treatment groups based upon these two data sets, with the differences between groups being assessed by a two-tailed Student’s t-test, using a P value<0.05 as the threshold for significance. The data for the post-treatment changes in blood flow, PpIX fluorescence, and tissue hypoxia were compared with the pretreatment value for each of the time points. Examples of the recorded images for the delayed fluorescence, prompt fluorescence, and blood flow are demonstrated in Figs. 1(c)–1(e).

## 3 Results

The PpIX concentration, tissue hypoxia, and the blood flow of each mouse in each group were recorded prior to the treatment, after the first fraction, after the 2-h dark period, after the second fraction, and 2 h post-PDT. Both hind legs were imaged to provide a measure of the PDT-treated leg and the contralateral control leg. The results of each treated leg were normalized to the control untreated leg of the same animal, as a way to provide data that could be effectively averaged across multiple animals.

**Fig. 2:**
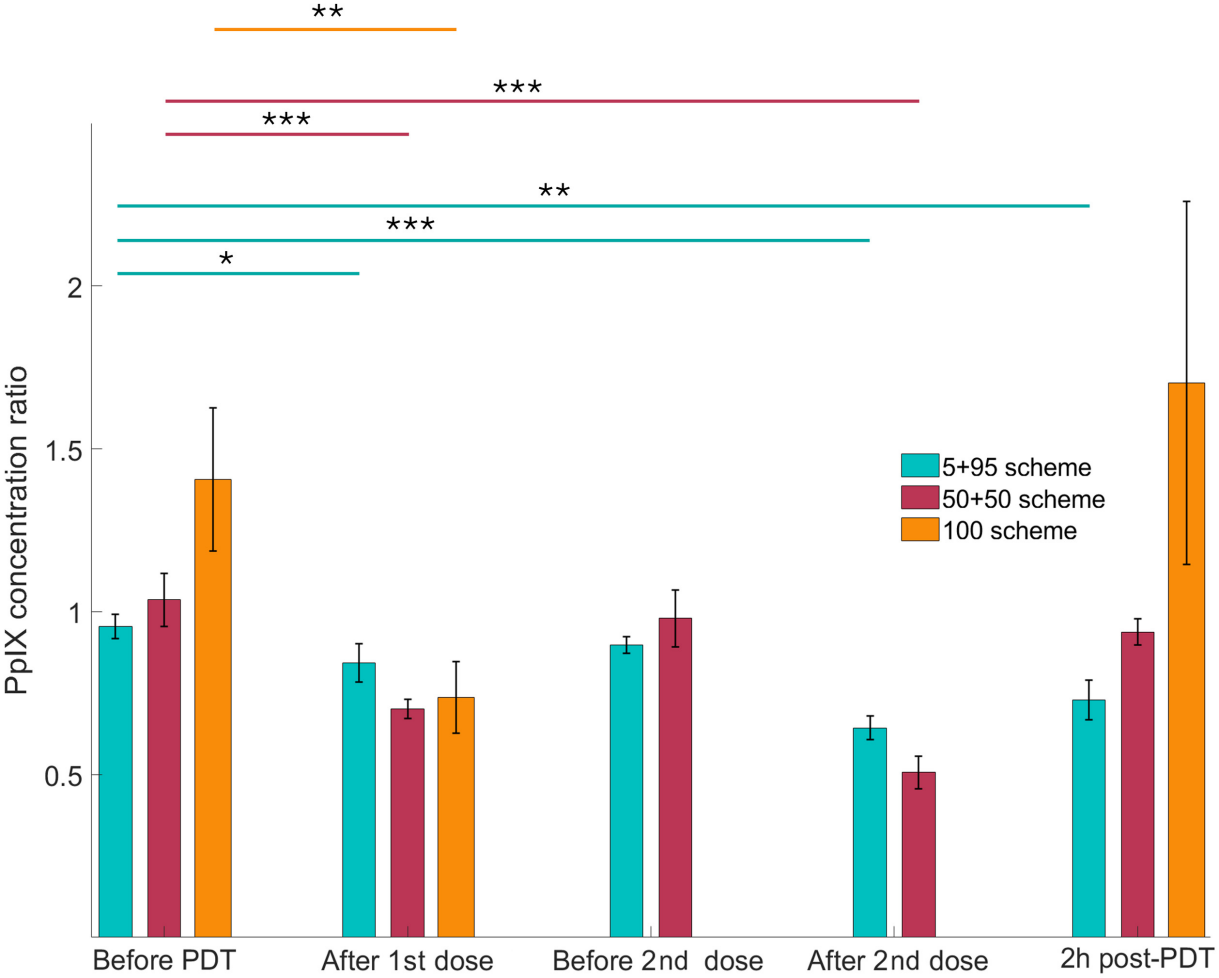
PpIX concentration ratio as measured by *in vivo* fluorescence: PDT leg/control leg for three fractionation schemes. Statistically significant differences in time for each group are denoted as *P<0.05, **P<0.01, and ***P<0.001. For this and next figure data, the post-PDT imaging was performed 2 h after the full dose was delivered in one or two fractions.

The PpIX prompt fluorescence data help estimate the PpIX concentration in the tissue and the amount of the photosensitizer replenishment during the dark periods between the PDT fractions. PpIX is known to photobleach during the PDT procedure due to the formation of photoproducts, which is manifested in lower concentration after the delivery of the PDT dose and, therefore, lower fluorescence intensity. This control of the photobleached PpIX is a proposed method to control the delivered PDT dose. After the initial 2-h PpIX production pretreatment, we observe the photobleaching after the first dose (or full dose in the case of the 100 schemes) deliveries. As shown in Fig. 2, the concentration of PpIX is returning to the pretreatment levels in the fractionated treatment schemes, allowing for to delivery of a second light dose and triggering a PDT effect by producing singlet oxygen in the tissue.

**Fig. 3:**
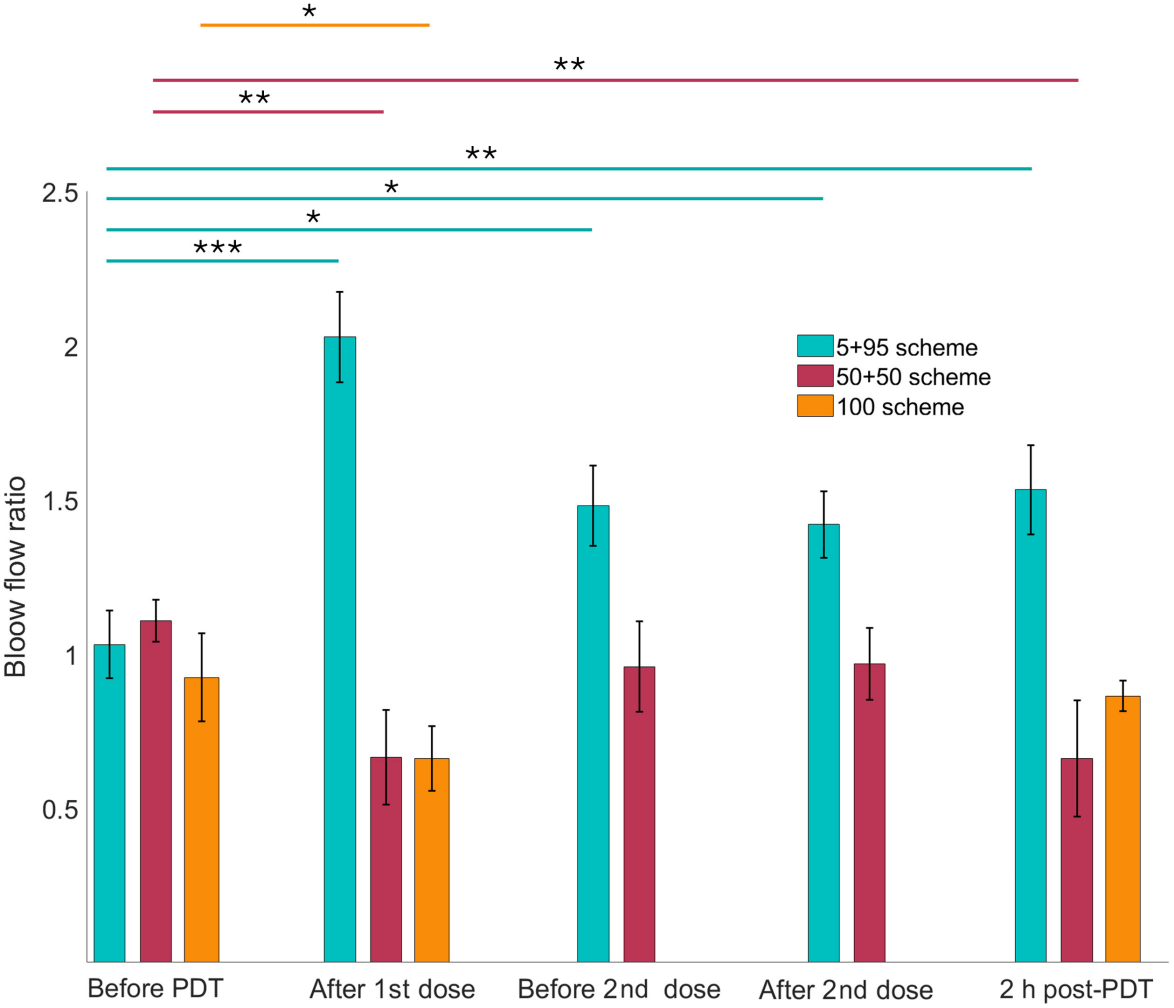
Doppler blood flow measurement ratio for the PDT leg/control leg for three treatment schemes. Statistically significant differences in time for each group are denoted as *P<0.05, **P<0.01, and ***P<0.001.

Figure 3 demonstrates the changes in the blood flow in the PDT-treated leg for all three treatment groups. We observe a significant increase in the PDT-treated leg after delivering 5% of the total dose. In contrast to that, the blood flow changes in the treated leg after delivery of 50% and 100% PDT doses demonstrate a noticeable decrease immediately after the treatment.

Out of three irradiation schemes used only the 5 + 95 fractionation of the PDT light results in vascular dilation, increasing the blood flow and facilitating the drug delivery and the oxygen supply to the treatment site. We demonstrate that even after the dark period of 2 h during which the PpIX is restored in the skin and the vascular vessel walls, the blood flow is significantly increased compared with the pretreatment state (P<0.05), and we continue to observe the vasodilation throughout the treatment even after the delivery of 95% of the PDT light.

The reduced blood flow observed after the treatment under both 50 + 50 and 100 schemes is a result of vascular constriction and even vascular damage, which slows down the oxygenation and the drug delivery, decreasing tissue permeability.

**Fig. 4:**
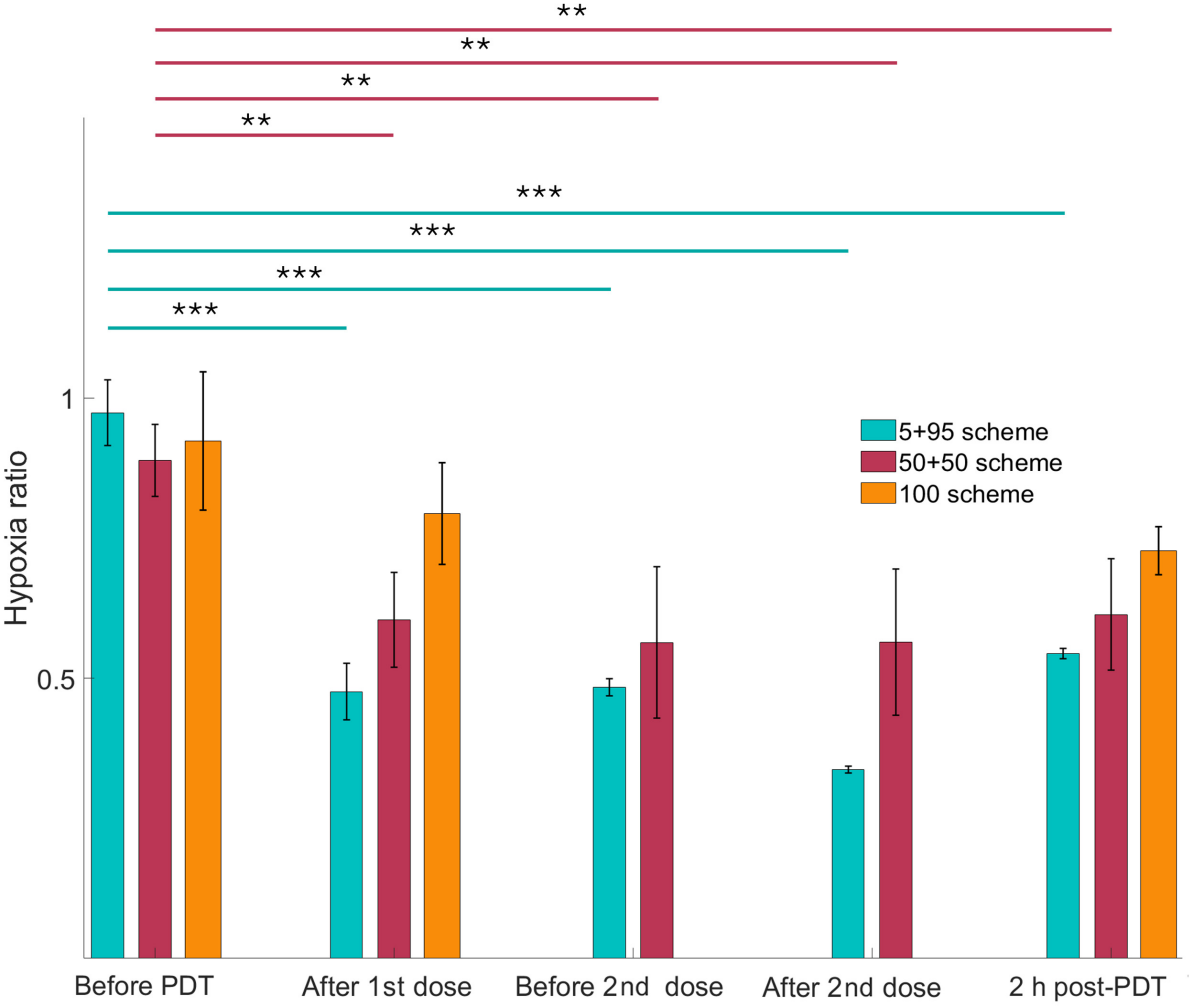
Hypoxia ratio as measured by delayed fluorescence for the PDT leg relative to the control leg. Statistically significant differences in time for each group are denoted as *P<0.05, **P<0.01, and ***P<0.001.

The changes in the tissue oxygenation were observed with the imaging of PpIX DF, which corresponds to tissue hypoxia. Figure 4 demonstrates the ratio of the PDT leg to the untreated leg’s hypoxia for all the irradiation schemes. We observe no statistically significant change in the tissue’s hypoxia after the total dose delivery in a single fraction. Interestingly, despite the pronounced vasoconstriction under the 50 + 50 treatment scheme, the oxygenation of the tissue increases (hypoxia signal decreases), although the change is more pronounced in the case of 5 + 95 fractionation.

**Fig. 5:**
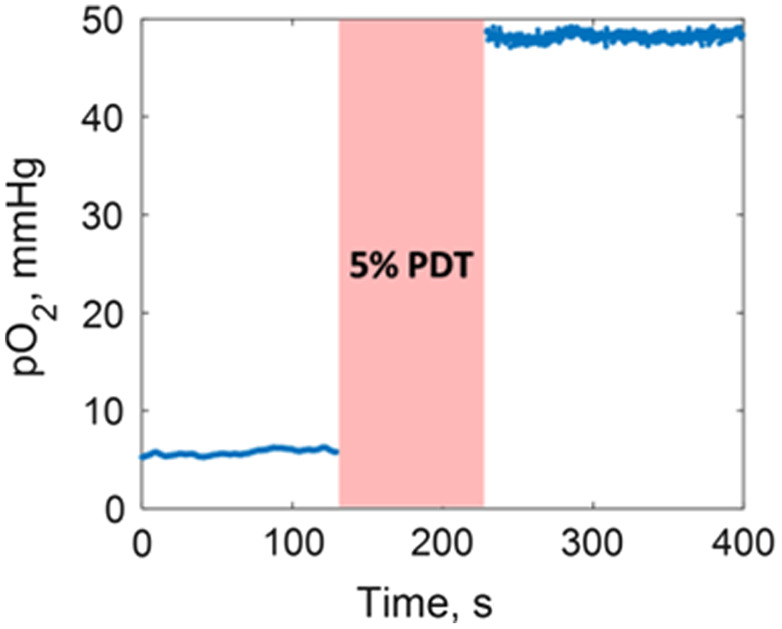
Measured ${\mathrm{pO}}_{2}$ of the treated leg of a mouse before and immediately after the delivery of the 5% PDT dose, showing a substantial rise in tissue ${\mathrm{pO}}_{2}$ after the 5% light delivery.

To confirm that oxygenation changes seen with delayed fluorescence from PpIX, a secondary oxygen measurement was taken by injection of Oxyphor PdG4 oxygen probe, and the tissue ${\mathrm{pO}}_{2}$ was directly measured before and after the delivery of 5% of the PDT dose. Figure 5 shows the change in the ${\mathrm{pO}}_{2}$ in the treated leg of one of the mice from the 5 + 95 scheme before and after the first dose delivery. We see that immediately after the irradiation, the tissue oxygenation is increased significantly, which aligns with the decrease of the hypoxia signal from the delayed fluorescence imaging of the 5 + 95 mice group. The other mice in this group demonstrated similar ${\mathrm{pO}}_{2}$ values.

## 4 Discussion and Conclusion

In this study, the concept of splitting the light dose and the use of a prepriming dose were examined in terms of the biophysical changes occurring in blood flow and oxygen supply. The three most-studied light fractionation schemes for the PDT treatment were examined as a starting point to understand the changes and their magnitudes. As previously demonstrated,^9^^–^^12^ the 5 + 95 scheme is known to cause the most PDT tissue damage, whereas the delivery of a single full 100% dose yields the lowest damage out of all three groups. Using this paradigm, the tissue hypoxia, blood flow, and PpIX concentration were each monitored. Measurements were done in both legs to allow for an internal control leg to compare against for each mouse. The data were normalized such that the measurements from the treated leg were divided by the untreated leg, as a way to minimize factors related to inter-animal biological variability. Both control and PDT leg had ALA solution applied to them to estimate the change in the PpIX concentration due to the photobleaching during the light irradiation. These controls and the diagnostic measurements performed were thought to be objective ways to quantify the changes occurring.

When comparing the three treatment schemes, observation showed that the case of the 5 + 95 fractionation scheme yielded the most PDT damage, as compared with the other schemes, as is known. In this treatment plan, the blood flow and the tissue oxygenation significantly increase, suggesting that the PDT damage occurring during treatment is actively being responded to by the body through things such as capillary dilation to increase flow. This phenomenon was observed not only after the delivery of the small dose but also after the delivery of the main portion of the PDT light as well.

The acute vascular response has been previously studied^41^ in the endothelial cells due to the ability of the dermal vasculature to generate PpIX. It has been demonstrated that a significant amount of PpIX is accumulated in and around endothelial cells, and the quick depletion of molecular oxygen results in a hypoxic environment. This oxygen depletion becomes faster with the increase in the fluence rate of the PDT light. In this study, the hypoxia signal is shown from intracellular measurements by the delayed PpIX fluorescence, which also increases with the increase of the delivered dose at the same fluence, manifesting faster oxygen depletion.

Becker et al.^42^ studied blood flow changes for different fluence rates ($75/35/10\;\mathrm{mW}/{\mathrm{cm}}^{2}$ with a light dose of $80/37.5\;J/{\mathrm{cm}}^{2}$). They demonstrated that the blood flow initially increased and then decreased during the treatment, and this change was fluence-dependent. These results are comparable to our observations of the blood flow decrease in 50 + 50 or 100 schemes. Schacht et al.^43^ also demonstrated a large decrease in the red blood cell velocity at a high dose of $100\;J/{\mathrm{cm}}^{2}$, but not at a low dose of $10\;J/{\mathrm{cm}}^{2}$. All these results are consistent with our observations.

The studies of the superficial vasculature photosensitization^16^ using different PpIX precursors demonstrate that after application of all three porphyrin precursors, PpIX was synthesized in the vessel walls, and it causes vascular damage. It was shown that the amount of PpIX in the endothelial cells of the vessel walls was related to the damage from the PDT treatment. The authors illustrated that the superficial vasculature is a target for ALA-PDT, and endothelial cells are particularly sensitive to ALA-PDT. Vascular damage after light-fractionated PDT might explain the increased blood volume in the 5 + 95 scheme that we observed in this study.

The *in vitro* studies on cell cultures also demonstrated the efficiency increase in PDT treatment when using the dose fractionation^44^ where the first dose would cause sublethal cell damage, which leads to an increased sensitivity to a second fraction. The authors suggest that the light fractionation enhances the cell response of the cell populations with enhanced PpIX production such as the local vasculature, thus enhancing clinical efficacy. The increase of blood volume in the treatment area that we observe in the 5 + 95 scheme (Fig. 3) is very likely contributing to the increase of the tissue oxygenation detected from the changes in ${\mathrm{pO}}_{2}$ and the tissue hypoxia from vascular damage. It appears that these two factors combined are contributing to the increase of the PDT^9^^–^^12^ when using the 5 + 95 fractionation scheme compared with the other fractionation PDT treatments or a full PDT dose delivery. The increased oxygenation of the tissue after the delivery of the first PDT dose continues throughout the entire treatment. Thus, it is likely to result in enhanced production of singlet oxygen in the treated tissue, which results in increased PDT damage from this fractionation treatment scheme.

The delivery of a small dose before the dark interval and the residual dose delivery act as a priming mechanism. In photodynamic therapy, photodynamic priming is a technique combining PDT with another treatment method, such as chemotherapy. It has been widely accepted that PDP affects chemotherapy, enhancing its efficacy. In case of the 5 + 95 scheme, the first 5% dose acts such as priming therapy for PDT, and the 2-h interval is required to replenish the PpIX in the tissue to be able to access the photosensitizer. Then, after the delivery of the residual dose, the tissue stays primed, significantly increasing the outcome of a regular PDT treatment.

These changes may be related to the recent observations of ALA being a radiation sensitizer as well, given that subtle amounts of light can induce PDT damage that leads to increased oxygenation.^45^^,^^46^ The systemic ALA administration^13^^–^^16^ also supports the role of the vasculature sensitization following the PDT treatment. It seems feasible that this priming effect of ALA-PDT may be a suitable neoadjuvant treatment for enhancing radiotherapy damage via higher oxygenation. These findings demonstrate that a small dose of PDT light can act as a priming mechanism significantly increasing the therapeutic efficiency of a following procedure without causing additional damage associated with PDT.

## Data Availability

Data underlying the results presented in this paper are not publicly available at this time but may be obtained from the authors upon reasonable request.
